# Buffalo Academy of Medicine

**Published:** 1904-01

**Authors:** Wm. Irving Thornton


### SOCIETY PROCEEDINGS.

### Buffalo Academy of Medicine

*Section on Medicine, November 10, 1903.*

Reported by WM. IRVING THORNTON, M. D., Secretary.

THE regular meeting of the medical section was held Tuesday evening, November 10, 1903, at the academy rooms in the Public Library Building. The meeting was called to order at 8.45 o'clock by the chairman, Dr. A. W. Hurd, whereupon the minutes of the last meeting were read and approved.

The chairman spoke of the death of Dr. Cornelius C. Wyckoff, and suggested that appropriate action be taken by the section. On motion, the chairman appointed as a committee to draft resolutions, Drs. Chas. S. Jones, A. W. Bayliss and H. G. Matzinger.

The paper of the evening was presented by Dr. Eugene Wasdin, entitled,

SOME CLINICAL OBSERVATIONS ON TYPHOID FEVER.

During the course of his remarks, charts from a number of cases of typhoid fever were presented, which tended to show the normal duration of pure uncomplicated typhoid to be 14 days, and in nearly all cases which extended longer than that period, a marked diminution of temperature was seen upon the fourteenth day. Cases lasting 21 days were explained upon the ground that two cycles, each of 14 days, existed overlapping one another; the second cycle beginning at the second week of the disease and due to a secondary or autoinfection of the body with the typhoid organisms. On account of the great frequency of bronchial and lung complications in typhoid, the mode of infection was considered to be by the respiratory tract, rather than through the gastrointestinal tract.

The paper was discussed by Drs. Rochester, Kenerson, Thoma and Ingraham.

The committee appointed to prepare resolutions upon the death of Dr. Wyckoff then reported as follows:

Whereas, Dr. Cornelius C. Wyckoff died November 7, 1903, at his residence in Buffalo, at the advanced age of 81 years and, after more than half a century of honorable, active service in the practice of his profession to the day of his death, was taken from our midst, the city of Buffalo thereby losing a loyal citizen, and the profession a respected member, whose devotion to his profession and his many friends we commend; therefore, be it

*Resolved,* That the section of medicine of the Buffalo Academy of Medicine expresses its appreciation of his honorable career and its sorrow at the loss of a devoted member, as well as its profound sympathy to the bereaved family.

The preamble and resolution were adopted, ordered spread upon the minutes and a copy directed to be sent to Mrs. Wyckoff.

The meeting adjourned at 10.10 p. m. Members present, 34.

